# Peginterferon plus Ribavirin for HIV-infected Patients with Treatment-Naïve Acute or Chronic HCV Infection in Taiwan: A Prospective Cohort Study

**DOI:** 10.1038/srep17410

**Published:** 2015-11-30

**Authors:** Chen-Hua Liu, Wang-Hui Sheng, Hsin-Yun Sun, Szu-Min Hsieh, Yi-Chun Lo, Chun-Jen Liu, Tung-Hung Su, Hung-Chih Yang, Wen-Chun Liu, Pei-Jer Chen, Ding-Shinn Chen, Chien-Ching Hung, Jia-Horng Kao

**Affiliations:** 1 Department of Internal Medicine, National Taiwan University Hospital and National Taiwan University College of Medicine, Taipei, Taiwan; 2 Hepatitis Research Center, National Taiwan University Hospital, Taipei, Taiwan; 3 Graduate Institute of Clinical Medicine, National Taiwan University College of Medicine, Taipei, Taiwan; 4 Department of Internal Medicine, National Taiwan University Hospital, Yun-Lin Branch, Douliou, Taiwan; 5 Office of Preventive Medicine, Centers for Disease Control, Taipei, Taiwan; 6 Department of Microbiology, National Taiwan University College of Medicine, Taipei, Taiwan; 7 Genomics Research Center, Academia Sinica, Taipei, Taiwan; 8 Department of Medical Research, China Medical University Hospital, Taichung, Taiwan; 9 China Medical University, Taichung, Taiwan

## Abstract

Data are limited on the effectiveness and safety of peginterferon plus ribavirin in HIV-infected Asian patients with acute or chronic HCV infection. HIV-infected Taiwanese patients with acute HCV infection received peginterferon plus weight-based ribavirin for 24 weeks (n = 24), and those with chronic HCV genotype 1 or 6 (HCV-1/6) and HCV genotype 2 or 3 (HCV-2/3) infection received response-guided therapy for 12–72 and 24–48 weeks, respectively (n = 92). The primary endpoint was sustained virologic response (SVR), defined as undetectable HCV RNA 24 weeks off-therapy. The SVR rates were 83% and 72% in patients with acute and chronic HCV infection (*p* = 0.30), and 68% and 72% in patients with chronic HCV-1/6 and HCV-2/3 infection (*p* = 0.48), respectively. While no factors predicted SVR in acute HCV and chronic HCV-2/3 infection, age (odds ratio [OR] per 1-year increase: 0.88, 95% confidence interval [CI]: 0.78–0.99, *p* = 0.04), HCV RNA (OR per 1-log_10_ increase: 0.18, 95% CI: 0.03–0.98, *p* = 0.03), *IL28B* genotype (OR: 5.52, 95% CI: 1.55–12.2, *p* = 0.02), and RVR (OR: 9.62, 95% CI: 3.89–15.3, *p* = 0.007) predicted SVR in chronic HCV-1/6 infection. In conclusion, the SVR rates of peginterferon plus ribavirin for 24 weeks and for response-guided 12–72 weeks are satisfactory in HIV-infected Taiwanese patients with acute and chronic HCV infection.

Due to the shared routes of transmission and lack of effective vaccination, hepatitis C virus (HCV) infection is the major comorbidity of patients with human immunodeficiency virus (HIV) infection. It is estimated that approximately 10 million people worldwide have HIV and HCV (HIV/HCV) coinfection^1^. HIV-infected patients with acute HCV infection have a higher risk of evolving to chronic HCV infection than those without HIV infection^2^,^3^. Once chronic HCV infection is established, patients with HIV/HCV coinfection tend to have higher serum HCV RNA levels, higher risks of maternal-to-fetal transmission, faster hepatic fibrosis progression, and higher liver-related morbidity and mortality, regardless of receiving highly active antiretroviral therapy (HAART) or not^4^,^5^,^6^,^7^,^8^. In contrast, patients with HIV/HCV coinfection have improved survival after successful HCV eradication^9^,^10^.

Over the past two decades, interferon (IFN)-based therapy has been used to treat acute and chronic HCV infections in patients with HIV coinfection. The sustained virologic response (SVR) rates in patients with acute HCV infection are 60–80% by 24 weeks of peginterferon plus weight-based ribavirin therapy^11^. However, the SVR rates in HIV-infected patients with chronic HCV infection by 24–48 weeks of combination therapy are only 27–50%^12^,^13^,^14^,^15^,^16^. Furthermore, patients with HCV genotype 1 or 4 (HCV-1/4) infection have lower SVR rates than those with genotype 2 or 3 (HCV-2/3) infection (14–38% versus 44–73%). Although HIV/HCV coinfected patients with rapid virologic response (RVR) may receive a truncated duration of peginterferon plus ribavirin on the basis of response-guided therapy, only 18–32% and 65% of them with HCV-1/4 and HCV-2/3 infection can meet the criteria^17^,^18^. Moreover, the SVR rates are even poorer in HIV/HCV coinfected patients with HCV-1 infection and high baseline viral load (>400,000–800,000 IU/mL)^12^,^18^,^19^,^20^.

Data are limited in HIV-infected patients with acute HCV infection receiving direct acting antiviral agents (DAAs). One study showed that the SVR rate was 84% in acute HCV-1 patients receiving telaprevir-based triple therapy^21^. The SVR rates were 63–74% in HIV-infected patients with chronic HCV-1 infection receiving boceprevir or telaprevir-based triple therapy^22^,^23^. Twelve to 24 weeks of IFN-free regimens with sofosbuvir plus ribavirin, ombitasvir/paritaprevir/ritonavir/dasabuvir plus ribavirin, grazoprevir/elbasvir plus ribavirin, or ledipasvir/sofosbuvir further increased the SVR rates to 76–97% in HIV-infected patients with chronic HCV-1 infection^24^,^25^,^26^,^27^. In addition, the SVR rates for HIV-infected patients with treatment-naive and treatment-experienced chronic HCV-2/3 infection were 75% and 93% by 12 and 24 weeks of sofosbuvir plus ribavirin therapy, respectively^24^. Although using DAAs with/without peginterferon plus ribavirin is highly effective for these patients, the high cost and limited access to these novel agents preclude their widespread use in resource-limiting or resource-poor countries.

Few studies evaluated the treatment of HCV in HIV-infected Asian patients. Two small studies in Japan evaluated the efficacy of peginterferon plus ribavirin in 12 and 10 HIV-infected patients with acute and chronic HCV infection, with an SVR rate of 75% and 60%, respectively^28^,^29^. Furthermore, one HIV-infected Japanese patient with chronic HCV-1 infection was successfully retreated by telaprevir-based therapy^30^. Considering the high prevalence of favorable interleukin-28B (*IL28B*) genotype which may predict a high rate of SVR to peginterferon plus ribavirin in Asians, we aimed to evaluate the effectiveness and safety of peginterferon plus weight-based ribavirin for HIV-infected patients with treatment-naïve acute or chronic HCV infections in Taiwan.

## Results

### Patient Characteristics

**Figure 1:**
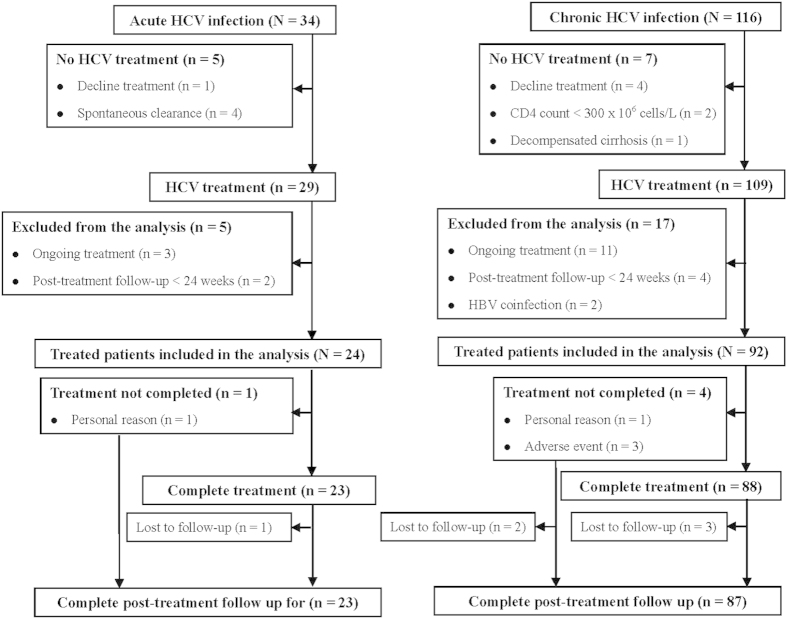
Flow diagram of HIV-infected patients with acute or chronic HCV infection according to treatment status.

Among the 34 patients with documented acute HCV infection, 5 did not receive treatment because of spontaneous viral clearance in 4 (11.8%) and decline for treatment in 1 (2.9%). Five of the 29 treated patients were excluded from the analysis because of ongoing treatment in 3 and post-treatment follow-up <24 weeks in 2. Among the 116 patients with chronic HCV infection, 7 did not receive treatment because of decline for treatment in 4, lymphopenia in 2, and decompensated cirrhosis in 1. Seventeen of the 109 treated patients were excluded from the analysis because of ongoing treatmentin 11, post-treatment follow-up <24 weeks in 4, and hepatitis B virus (HBV) coinfection in 2. Finally, 24 and 92 HIV-infected patients with acute and chronic HCV infections were included in the study, respectively (Fig. 1).

Compared to patients with acute HCV infection, those with chronic HCV infection had higher baseline HCV viral load, more advanced hepatic fibrosis, and lower serum alanine aminotransferase (ALT) quotient (Table 1). The baseline characteristics for patients with chronic HCV-1/6 and HCV-2/3 infection were similar, except for a higher proportion of injection drug users (IDUs) in HCV-1/6 patients.

Of patients included in the analysis, 23 of 24 (95.8%) and 88 of 92 (95.7%) patients with acute and chronic HCV infection complete the assigned treatment, and 23 of 24 (95.8%) and 87 of 92 (94.6%) of them completed the post-treatment follow-up for 24 weeks, respectively. One patient with acute HCV infection prematurely stopped treatment at week 16 due to personal reason. Furthermore, 1 and 3 patients with chronic HCV infection prematurely stopped treatment at weeks 20 (Arm F), 32 (Arm G), 36 (Arm B) and 64 (Arm D) due to personal reason and treatment-emergent adverse events (TEAEs), respectively. Four patients who completed treatment (1 with acute HCV infection; 3 with chronic HCV infection in arms C, F, and G) and another 2 who prematurely stopped treatment (Arms D and G) did not complete post-treatment follow-up for 24 weeks. However, all these 6 patients had undetectable HCV RNA at the end-of-treatment (Fig. 1).

### Effectiveness

Compared to patients with chronic HCV infection, those with acute HCV infection had a greater RVR rate (100% versus 54%, *p* < 0.001). The early virologic response (EVR) (100% versus 96%, *p* = 0.58), end-of-treatment virologic response (ETVR) (96% versus 93%, *p* = 0.99), and SVR (83% versus 72%, *p* = 0.30) were comparable between patients with acute and chronic HCV infection (Table 2). Patients with chronic HCV-2/3 infection had greater RVR (72% versus 41%, *p* = 0.006) and ETVR (100% versus 89%, *p* = 0.04) rates than those with chronic HCV-1/6 infection. The EVR (92% versus 100%, *p* = 0.13) and SVR (68% versus 77%, *p* = 0.48) rates were comparable between patients with chronic HCV-1/6 and HCV-2/3 infection. The SVR rates were 92%, 90%, 75%, 36% and 0% in chronic HCV-1/6 Arms A-E, and 82% and 64% in chronic HCV-2/3 Arms F & G, respectively (Table 3).

### Safety

The on-treatment constitutional and common laboratory AEs in patients with acute or chronic HCV infection are shown in Table 4. The rates of constitutional TEAEs were comparable among patients receiving different treatment regimens, except for higher rates of hair loss/alopecia in patients with longer durations of therapy. The rates of anemia and leukopenia in patients receiving 48–72 weeks of therapy were higher than those receiving 12–24 weeks of therapy. However, the rates of thrombocytopenia were comparable among various treatment arms.

### Prespecified Factors to Predict SVR

By univariate analysis, age (*p* = 0.05) and HCV RNA level (*p* = 0.07) were associated with SVR in patients with acute HCV infection. Multivariate analysis showed that no factors could predict SVR in these patients. In patients with chronic HCV infection, age (*p* = 0.01), HIV risk behavior (*p* = 0.09), HIV RNA level (*p* = 0.02), cluster of differentiation 4 (CD4) count (*p* = 0.006), HCV RNA level (*p* = 0.008), *IL28B* genotype (*p* = 0.004), and RVR (*p* = 0.006) were associated with SVR by univariate analysis. Multivariate analysis showed that older age (odds ratio [OR] per-1-year increase: 0.91, 95% confidence interval [CI]: 0.81–0.99, *p* = 0.03), higher HCV RNA level (OR per 1-log_10_ increase: 0.35, 95% CI: 0.14–0.84, *p* = 0.02), favorable *IL28B* genotype (OR: 5.56, 95% CI: 2.39–8.33, *p* = 0.006), and the presence of RVR (OR: 8.33, 95% CI: 5.89–14.3, *p* = 0.003) were independent predictors for SVR (Table 5).

In patients with chronic HCV-1/6 infection, univariate analysis showed that age (*p* = 0.005), CD4 count (*p* = 0.08), HCV RNA level (*p* = 0.006), *IL28B* genotype (*p* = 0.03), and RVR (*p* = 0.003) were associated with SVR. Multivariate analysis of these factors showed that older age (OR per 1-year increase: 0.88, 95% CI: 0.78–0.99, *p* = 0.04), higher HCV RNA level (OR per 1-log_10_ increase: 0.18, 95% CI: 0.03–0.98, *p* = 0.03), favorable *IL28B* genotype (OR: 5.52, 95% CI: 1.55–12.2, *p* = 0.02), and the presence of RVR (OR: 9.62, 95% CI: 3.89–15.3, *p* = 0.007) were independent predictors for SVR. In patients with chronic HCV-2/3 infection, univariate analysis showed that CD4 count (*p* = 0.05) and *IL28B* genotype (*p* = 0.07) were associated with SVR. Multivariate analysis showed that no factors could predict SVR (Table 6).

## Discussion

In this prospective study, we evaluated the effectiveness and safety of peginterferon plus weight-based ribavirin in a sizable number of HIV-infected Asian patients with treatment-naïve acute or chronic HCV infection. The SVR rates of HIV-infected Taiwanese patients with acute and chronic HCV infections tended to be greater than HIV-infected Western patients (83% versus 60–80% and 72% versus 27–50%, respectively)^11^,^12^,^13^,^14^,^15^,^16^. By applying the response-guided therapy, the SVR rates could reach 68% and 77% in our HIV-infected patients with chronic HCV−1/6 and HCV-2/3 infections, respectively. In addition, the safety profiles in our patients were comparable to those in Western patients^11^,^12^,^13^,^14^,^15^,^16^.

HCV infection among HIV-infected patients remains a major health problem in Taiwan. A recent survey in Taiwan revealed that the incidence rate of acute HCV infection was 7.03 per 1,000 person-years, and the rates tended to increase from 1994 to 2010^31^. In line with previous reports, we found that as high as 92% of our HIV-infected patients were men who have sex with men (MSM), and the spontaneous viral clearance rate following acute HCV infection was only 11.8%^11^,^21^,^28^,^31^. Because the rates of incidence and evolution to chronicity are high in HIV-infected patients with acute HCV infection, the prevalence rates of chronic HCV infection were 96.8% among HIV-infected IDUs and 6.4% among those who acquired HIV through sexual routes in Taiwan^32^,^33^. Therefore, early identification and treatment of HCV infection are important to improve the clinical outcome in HIV-infected patients.

The RVR and EVR rates were excellent (100%) in our patients with acute HCV infection. Although one patient had viral breakthrough at the end-of-treatment, the SVR rate was 83% with 24 weeks of peginterferon plus ribavirin therapy, which was comparable to that of HIV-infected Japanese patients receiving the same regimen, and to that of HIV-negative individuals receiving 12–24 weeks of peginterferon monotherapy for acute HCV infection^28^,^34^,^35^. Furthermore, no patients prematurely discontinued treatment due to TEAEs. Although not statistically significant, our findings showed that age and baseline HCV RNA level tended to be associated with SVR^28^. The effectiveness and safety profiles in our study suggest that HIV-infected Taiwanese patients with acute HCV infection should receive peginterferon plus ribavirin for 24 weeks if they have no spontaneous viral clearance after 12 weeks of observation^36^.

Compared to patients with acute HCV infection, those with chronic HCV infection had lower ALT levels, higher baseline HCV RNA levels, and more advanced hepatic fibrosis, which have been shown to be associated with decreased responses to peginterferon plus ribavirin^12^,^13^,^14^,^15^. However, our patients with chronic HCV infection who received 12–72 weeks of response-guided therapy reached an SVR rate of 72%, which was comparable to our patients with acute HCV infection (83%), and Spanish patients with chronic HCV infection who received 24–60 weeks of response-guided therapy (67%)^17^. Furthermore, only 3 of 92 (3.3%) patients prematurely discontinued treatment due to TEAEs. Applying response-guided peginterferon plus ribavirin therapy is safe and effective for HIV-infected Taiwanese patients with chronic HCV infection.

Our patients with chronic HCV-1/6 and HCV-2/3 infections had greater RVR rates than HIV-infected Western patients (41% versus 18–32% and 72% versus 65%), which could be reasoned by the higher prevalence of favorable *IL28B* genotype in Asian populations^37^,^38^. However, the RVR rates of our patients were lower than those of HIV-negative Asian patients with chronic HCV-1 (54–55%), HCV-6 (77–82%) and HCV-2 (87%) infection, implying that HIV coinfection was an adverse factor of on-treatment viral decline^39^,^40^,^41^. By using the same response-guided therapy, the SVR rates in our patients were slightly inferior to those in HIV-negative Asian patients with chronic HCV-1/6 (92% versus 94%, 90% versus 95%, 75% versus 85%, and 36% versus 50% in Arms A-D) and HCV-2/3 (82% versus 98%, and 64% versus 70% in Arms F and G) infection (Table 3)^39^,^42^,^43^. Our findings suggest that HIV-infected patients with chronic HCV infection can achieve comparable SVR rates to HCV-monoinfected patients if they receive individualized peginterferon plus ribavirin therapy.

We further analyzed the prespecified factors predictive of SVR. In contrast to patients with chronic HCV infection where age, HCV RNA level, *IL28B* genotype, and RVR independently predicted SVR, none of them could predict SVR in patients with acute HCV infection^11^,^28^,^35^. Because the SVR rate was relatively high in our patients with acute HCV infection and no negative factors were associated with SVR, the physicians should encourage these patients to receive treatment if they fail to spontaneously clear HCV after 12 weeks of observation. While age, HCV RNA level, *IL28B* genotype, and RVR independently predicted SVR in our patients with chronic HCV-1/6 infection, no factors could predict SVR in our patients with chronic HCV-2/3 infection^17^,^18^,^20^,^37^,^38^,^39^,^40^,^41^,^42^. Our findings suggest that HIV-infected patients with chronic HCV-2/3 patients can achieve good SVR rates by response-guided therapy even they have unfavorable prespecified factors^43^,^44^.

Although the overall SVR rates in our patients with acute or chronic HCV infection were satisfactory, the response rates were significantly reduced in patients with chronic HCV infection who did not achieve RVR. Considering the limited availability and affordability of DAA in many Asian countries, these agents should be reserved for treatment-naïve HIV-infected Asian patients who are ineligible for or fail to achieve RVR by peginterferon plus ribavirin therapy, and for those who are intolerant, or fail prior peginterferon plus ribavirin therapy^45^,^46^.

In conclusion, the SVR rates are satisfactory in HIV-infected Taiwanese patients with treatment-naïve acute and chronic HCV infection receiving fixed duration 24 weeks and response-guided 12–72 weeks of peginterferon plus ribavirin, respectively.

## Methods

### Patients

This was a prospective cohort study, including a total of 150 HIV-infected Taiwanese patients aged ≥20 years with treatment-naïve acute or chronic HCV infection who were evaluated for peginterferon plus ribavirin therapy at National Taiwan University Hospital (NTUH) and NTUH Yun-Lin Branch between 2009 and 2014. Acute HCV infection was defined as the documentation of anti-HCV seroconversion (Abbott HCV EIA 3.0, Abbott Laboratories, Abbott Park, Illinois, USA), abrupt increase of the serum ALT levels **≥**10 times the upper limit of normal (ULN), and the presence of detectable serum HCV RNA (Cobas TaqMan HCV Test v2.0, Roche Diagnostics GmbH, Mannheim, Germany, limit of quantification: 25 IU/mL) within 6 months^47^. Chronic HCV infection was defined as presence of anti-HCV antibody and serum HCV RNA for ≥6 months.

The study was approved by NTUH Institutional Review Board and was conducted in accordance with the principles of Declaration of Helsinki and the International Conference on Harmonization for Good Clinical Practice. The informed consent was obtained from all subjects who participated in the study.

### Study Design

Baseline demographic data, hemogram, biochemical assays (serum albumin, bilirubin, ALT, and creatinine), anti-HCV, HBV surface antigen (HBsAg), anti-HIV, HCV RNA, HIV RNA, HCV genotype (Versant LiPA v2.0, Siemens Healthcare Diagnostics, IL, USA) and human genomic assay for *IL28B* rs8099917 genotypes (ABI TaqMan allelic discrimination kit and ABI7900HT Sequence Detection System, Applied Biosystems, Life Technologies Corporation, Grand Island, New York, USA) were evaluated before treatment^42^,^48^. The stage of hepatic fibrosis, which was assessed by transient elastography (Fibroscan®, Echosens, France), was determined as follows: F0 (<6.0 kPa), F1 (6.0–7.1 kPa), F2 (7.2–9.4 kPa), F3 (9.5–14.5 kPa), and F4 (≥14.6 kPa)^49^. The baseline viral load for HCV was defined as a low or high level with a cutoff value of 800,000 IU/mL^20^. Favorable *IL28B* rs8099917 genotype was defined as patients with homozygous TT genotype, whereas unfavorable genotype was defined as patients with heterozygous GT or homozygous GG genotype. Significant hepatic fibrosis was defined as a fibrotic stage of ≥F2 by METAVIR score.

For patients who declined treatment or who were not eligible for treatment (patients with hemoglobin levels <13 g/dL for men or <12 g/dL for women; absolute neutrophil count <1,500 × 10^6^ cells/L; platelet count <90 × 10^9^ cells/L; CD4 count <300 × 10^6^ cells/L; heavy alcohol use [alcohol consumption >20 g/day]; serum creatinine level ≥1.5 times ULN; decompensated cirrhosis; pregnancy; poorly controlled autoimmune diseases, cardiopulmonary diseases, neuropsychiatric diseases, and diabetes mellitus with retinopathy), they were excluded from the study.

**Figure 2:**
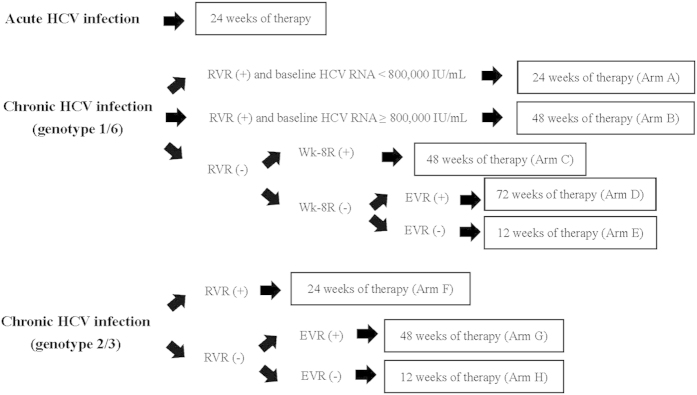
Treatment algorithms in HIV-infected patients with acute or chronic HCV infection by peginterferon alfa-2a plus weight-based ribavirin*. HIV, human immunodeficiency virus; HCV, hepatitis C virus; RVR, rapid virologic response; Wk-8R, week 8 virologic response; EVR, early virologic response. *Peginterferon alfa-2a at a dosage of 180 μg/week; ribavirin at a dosage of 1,000–1,200 mg/day (cut-off body weight, 75 kg).

All treated patients received peginterferon alfa-2a 180 μg/week (Pegasys, Hoffman-LaRoche, Basel, Switzerland) plus weight-based ribavirin (Copegus, Hoffman-LaRoche, Basel, Switzerland; 1,000 mg/day for patients weighted <75 kg and 1,200 mg/day for patients weighted ≥75 kg). Because patients with acute HCV infection may spontaneously clear viremia, they received follow-up for 12 weeks before the decision to initiate treatment. If patients had persistent viremia for ≥12 weeks, they received 24 weeks of treatment. Patients with chronic HCV-1/6 infection were treated for 24 weeks if they had low baseline viral load and achieved RVR (defined as undetectable serum HCV RNA at week 4 of treatment); 48 weeks if they had high baseline viral load and achieved RVR, or if they failed to achieve RVR but achieved week 8 virologic response (Wk-8R, defined as undetectable serum HCV RNA at week 8 of treatment in the absence of RVR); 72 weeks of they failed to achieved Wk-8R but achieved EVR (defined as ≥2 log_10_ viral load reduction from baseline to week 12 of treatment)(Arms A-D)^20^,^42^. Patients with chronic HCV-2/3 infection, they were treated for 24 weeks of they achieved RVR; 48 weeks if they failed to achieve RVR (Arms F and G)^43^. All patients received 12 weeks of treatment if they failed to achieve EVR (Arms E and H)(Fig. 2). Patients with mixed HCV genotypes 1/2 and 1/6a or with HCV genotypes 2/3 infections received the same response-guided therapies as proposed for those with HCV-1/6 or HCV-2/3monoinfection. For TEAEs, the dosages of peginterferon and ribavirin were adjusted according to package insert’s recommendations.

In patients who had received anti-HCV treatment, they were excluded from the analysis if they were still on-treatment, had not received post-treatment follow-up for 24 weeks to determine the off-therapy responses, or were co-infected with HBV.

### Effectiveness

On-treatment serum HCV RNA levels were assessed at weeks 4, 12 and 24 for acute HCV infection. In addition, the on-treatment serum HCV RNA levels were assessed at weeks 4, 8, 12, 24, 48 and 72 according to the proposed treatment duration for chronic HCV infection. For patients who prematurely discontinued treatment, the ETVR was assessed at the time of treatment discontinuation.

The primary endpoint was SVR, defined as patients with documented undetectable serum HCV RNA 24 weeks after the cessation of treatment^50^. Patients who had on-treatment viral breakthrough or who failed to achieve EVR were considered failure to achieved SVR, regardless of the availability of post-treatment serum HCV RNA data. Patients who relapsed after the treatment cessation and who lacked data to assess SVR were also considered failure to achieved SVR.

### Safety

The patients’ safety profiles, including the constitutional and laboratory AEs, were reported by a prespecified checklist. The grades of all AEs were assessed by the Common Terminology Criteria for Adverse Events (CTCAE), version 3.0.

### Statistical Analyses

Data were analyzed using Statistical Program for Social Sciences (SPSS 17.0; SPSS Inc., Chicago, Illinois, USA). Patient characteristics were expressed as mean (standard deviation, SD) and percentage when appropriate.

Baseline characteristics, on-treatment and off-therapy virologic responses between patients with acute and chronic HCV infections were compared by χ^2^ test, Fisher’s exact test, or two-sample *t*-test when appropriate. The relatedness of baseline characteristics and week 4 on-treatment virologic responses to SVR was analyzed by univariate analysis. Factors with a *p* value <0.10 by univariate analysis entered multivariate analysis to identify independent predictors for SVR, which were expressed by OR with 95% CI. All statistical tests were two-tailed and the results were statistically significant when a *p* value was <0.05.

## Additional Information

**How to cite this article**: Liu, C.-H. *et al.* Peginterferon plus Ribavirin for HIV-infected Patients with Treatment-Naïve Acute or Chronic HCV Infection in Taiwan: A Prospective Cohort Study. *Sci. Rep.* **5**, 17410; doi: 10.1038/srep17410 (2015).

## Figures and Tables

**Table 1:** Patient characteristics.

Variables*	All patients (N = 116)			Chronic HCV infection (N = 92)		*p* value
	Acute HCV infection (N = 24)	Chronic HCV infection (N = 92)	*p* value	Genotype 1/6 (n = 53)	Genotype 2/3 (n = 39)	
Mean age (SD), y	35 (8)	38 (8)	0.15	38 (8)	37 (8)	0.48
Sex			0.99			0.99
Male	22 (92)	84 (91)		48 (91)	36 (92)	
Female	2 (8)	8 (9)		5 (9)	3 (8)	
Mean hemoglobin level (SD), g/dL	15.6 (1.3)	15.1 (1.3)	0.18	14.8 (1.3)	15.4 (1.2)	0.12
Mean white blood cell count (SD), 10^6^ cells/L	5,555 (1,456)	6,064 (1,732)	0.19	6,148 (1,748)	5,951 (1,728)	0.59
Mean platelet count (SD), 10^9^ cells/L	239 (63)	210 (68)	0.06	211 (64)	208 (74)	0.83
Mean albumin level (SD), g/L	4.7 (0.2)	4.6 (0.4)	0.60	4.5 (0.4)	4.6 (0.4)	0.30
Mean total bilirubin level (SD), mg/dL	1.2 (0.6)	1.2 (0.9)	0.88	1.1 (0.8)	1.2 (1.0)	0.47
Mean ALT quotient (SD)	7.3 (6.7)	3.3 (3.2)	<0.001	3.2 (3.0)	3.4 (3.1)	0.27
HIV risk behavior			0.06			0.03
IDU	2 (8)	27 (29)		20 (38)	7 (18)	
MSM	22 (92)	62 (67)		30 (57)	32 (82)	
Hemophilia	0 (0)	3 (3)		3 (6)	0 (0)	
On HAART	23 (96)	83 (90)	0.69	49 (92)	34 (87)	0.49
Mean HIV RNA (SD), copies/mL	5,550 (26,934)	3,972 (15,294)	0.71	3,976 (15,137)	3,967 (15,704)	0.99
HIV-RNA <50 copies/mL	18 (75)	58 (63)	0.34	34 (64)	24 (62)	0.83
Mean CD4 cell count (SD), 10^6^ cells/L	439 (92)	428 (85)	0.21	415 (72)	445 (99)	0.10
HCV RNA (SD), log_10_ IU/mL	5.00 (1.45)	6.16 (1.36)	<0.001	6.36 (1.16)	5.90 (1.60)	0.11
HCV RNA ≥800,000 IU/mL	8 (33)	64 (70)	0.002	38 (72)	26 (67)	0.65
HCV genotype			0.61			<0.001
1	10 (42)	37 (40)		37 (70)	0 (0)	
1a	1 (4)	9 (10)		9 (17)	0 (0)	
1b	9 (38)	28 (30)		28 (53)	0 (0)	
2	12 (50)	34 (37)		0 (0)	34 (87)	
2a	12 (50)	30 (33)		0 (0)	30 (77)	
2b	0 (0)	4 (4)		0 (0)	4 (10)	
3a	0 (0)	4 (4)		0 (0)	4 (10)	
6a	1 (4)	8 (9)		8 (15)	0 (0)	
Mixed†	1 (4)	9 (10)		8 (15)	1 (3)	
1a + 1b	1 (4)	2 (2)		2 (4)	0 (0)	
1 + 2	0 (0)	4 (4)		4 (8)	0 (0)	
1 + 6a	0 (0)	2 (2)		2 (4)	0 (0)	
2 + 3a	0 (0)	1 (1)		0 (0)	1 (3)	
*IL28B* rs8099917 genotype			0.80			0.45
TT	19 (79)	67 (73)		36 (68)	31 (79)	
GT	4 (17)	21 (23)		14 (26)	7 (18)	
GG	1 (4)	4 (4)		3 (6)	1 (3)	
METAVIR fibrosis stage‡			0.005			0.47
F0	15 (63)	22 (24)		11 (21)	11 (28)	
F1	6 (25)	26 (28)		15 (28)	11 (28)	
F2	2 (8)	22 (24)		15 (28)	7 (18)	
F3	1 (4)	13 (14)		9 (17)	4 (10)	
F4	0 (0)	8 (9)		3 (6)	5 (13)	
Undetermined	0 (0)	1 (1)		0 (0)	1 (3)	

SD, standard deviation; ALT, alanine aminotransferase; HIV, human immunodeficiency virus; IDU, injection drug user; MSM, men who have sex with men; HAART, highly active antiretroviral therapy; CD, cluster of differentiation; RNA, ribonucleic acid; HCV, hepatitis C virus; *IL28B*, interleukin-28B.

^*^Values are numbers (percentages) unless otherwise indicated.

^†^Patients with mixed genotypes 1/2, or 1/6a infections were treated by the same protocol as those with genotype 1/6 monoinfection.

^‡^The stage of fibrosis was assessed by transient elastography (Fibroscan®, Echosens, Paris), and was determined according to the cut-off values proposed by Castera, *et al*^49^.

**Table 2:** Virologic responses in HIV-infected patients with acute or chronic HCV infection.

Virologic response*	Acute HCV infection (N = 24)	Chronic HCV infection(N = 92)	*p* value
RVR	24 (100)	50 (54)	<0.001
EVR	24 (100)	88 (96)	0.58
ETVR	23 (96)	86 (93)	0.99
SVR	20 (83)	66 (72)	0.30
Non-SVR	4 (17)	26 (27)	
Relapse	2 (8)	15 (16)	
No response	0 (0)	4 (4)	
Breakthrough	1 (4)	2 (2)	
Undetermined	1 (4)	5 (5)	

HIV, human immunodeficiency virus; HCV, hepatitis C virus; RVR, rapid virologic response; EVR, early virologic response; ETVR, end-of-treatment virologic response; SVR, sustained virologic response.

^*^Values are numbers (percentages).

**Table 3:** Virologic responses in HIV-infected patients with chronic HCV genotype 1/6 or genotype 2/3 infection.

Virologic response*	Genotype 1/6 (n = 53)						Genotype 2/3 (n = 39)				p value†
	Arm A (n = 12)	Arm B (n = 10)	Arm C (n = 16)	Arm D (n = 11)	Arm E (n = 4)	All (A-E) (n = 53)	Arm F (n = 28)	Arm G (n = 11)	Arm H (n = 0)	All (F-H) (n = 39)	
RVR	12 (100)	10 (100)	0 (0)	0 (0)	0 (0)	22 (41)	28 (100)	0 (0)	—	28 (72)	0.006
Wk-8R	—	—	16 (100)	0 (0)	0 (0)	—	—	—	—	—	—
EVR	12 (100)	10 (100)	16 (100)	11 (100)	0 (0)	49 (92)	28 (100)	11 (100)	—	39 (100)	0.13
ETVR	12 (100)	10 (100)	15 (94)	10 (91)	0 (0)	47 (89)	28 (100)	11 (100)	—	39 (100)	0.04
SVR	11 (92)	9 (90)	12 (75)	4 (36)	0 (0)	36 (68)	23 (82)	7 (64)	—	30 (77)	0.48
Non—SVR	1 (8)	1 (10)	4 (25)	7 (64)	4 (100)	17 (32)	3 (11)	6 (55)	—	9 (23)	
Relapse	1 (8)	1 (10)	2 (13)	5 (45)	0 (0)	9 (17)	2 (7)	4 (36)	—	6 (15)	
No response	0 (0)	0 (0)	0 (0)	0 (0)	4 (100)	4 (8)	0 (0)	0 (0)	—	0 (0)	
Breakthrough	0 (0)	0 (0)	1 (6)	1 (9)	0 (0)	2 (4)	0 (0)	0 (0)	—	0 (0)	
Undetermined	0 (0)	0 (0)	1 (6)	1 (9)	0 (0)	2 (4)	1 (4)	2 (18)	—	3 (8)	

HIV, human immunodeficiency virus; HCV, hepatitis C virus; RVR, rapid virologic response; Wk-8R, week 8 virologic response; EVR, early virologic response; ETVR, end-of-treatment virologic response; SVR, sustained virologic response.

^*^Values are numbers (percentages).

^†^Comparison for patients with HCV genotype 1/6 and HCV genotype 2/3 infections.

**Table 4:** Adverse events in HIV-infected patients with acute or chronic HCV infection.

Parameter*	Acute HCV infection	Chronic HCV infection						
	All (N = 24)	Arm A (N = 12)	Arm B (N = 10)	Arm C (N = 16)	Arm D (N = 11)	Arm E (N = 4)	Arm F (N = 28)	Arm G (N = 11)
Flu-like symptoms	7 (29)	4 (33)	3 (30)	4 (25)	4 (36)	2 (50)	9 (32)	3 (27)
Fatigue	6 (25)	4 (33)	4 (40)	6 (38)	2 (22)	1 (25)	8 (29)	2 (22)
Headache	2 (8)	1 (8)	1 (10)	1 (6)	1 (9)	0 (0)	3 (11)	1 (9)
Insomnia	6 (25)	4 (33)	3 (30)	4 (25)	3 (27)	1 (25)	9 (32)	2 (18)
Irritability	1 (4)	1 (8)	1 (10)	2 (13)	1 (9)	0 (0)	3 (11)	1 (9)
Depression	1 (4)	0 (0)	1 (10)	1 (6)	0 (0)	0 (0)	2 (7)	0 (0)
Anorexia	4 (17)	2 (17)	1 (10)	2 (13)	2 (22)	1 (25)	5 (18)	2 (18)
Diarrhea	1 (4)	0 (0)	0 (0)	1 (6)	1 (9)	0 (0)	2 (7)	0 (0)
Cough	1 (4)	0 (0)	0 (0)	1 (6)	1 (9)	0 (0)	2 (7)	0 (0)
Oral ulcer	2 (8)	1 (8)	1 (10)	1 (6)	1 (9)	1 (25)	3 (11)	1 (9)
Dermatitis	3 (13)	3 (25)	3 (30)	3 (19)	2 (22)	1 (25)	7 (25)	3 (27)
Injection site reaction	1 (4)	0 (0)	0 (0)	1 (6)	0 (0)	0 (0)	1 (4)	1 (9)
Hair loss/alopecia	2 (8)	1 (8)	1 (10)	3 (19)	2 (22)	0 (0)	3 (11)	2 (22)
Anemia†	3 (13)	2 (17)	3 (30)	4 (25)	4 (36)	0 (0)	5 (18)	3 (27)
Leukopenia‡	2 (8)	1 (8)	2 (20)	3 (19)	3 (27)	0 (0)	3 (11)	2 (22)
Thrombocytopenia§	0 (0)	0 (0)	0 (0)	1 (6)	0 (0)	0 (0)	2 (7)	0 (0)

^*^Values are numbers (percentages).

^†^Anemia was defined as a nadir hemoglobin level <10 g/dL.

^‡^Leukopenia was defined as a nadir absolute neutrophil count (ANC) <750 × 10^6^ cells/L

^§^Thrombocytopenia was defined as a nadir platelet count <50 × 10^9^ cells/L.

**Table 5:** Factors predictive of SVR in HIV-infected patients with acute or chronic HCV infection.

Variables*	Acute HCV infection (N = 24)					Chronic HCV infection (N = 92)				
	Univariate analysis			Multivariate analysis†		Univariate analysis			Multivariate analysis†	
	VR (n = 20)	Non-SVR (n = 4)	*p* value	OR (95% CI)	*p* value	SVR (n = 66)	Non-SVR (n = 26)	*p* value	OR (95% CI)	*p* value
Mean age (SD), y	33 (5)	41 (15)	0.05	0.85 (0.73–1.01)	0.07	36 (7)	41 (10)	0.01	0.91 (0.81–0.99)	0.03
Sex (male vs. female)	19/1 (95/5)	3/1 (75/25)	0.31	—	—	61/5 (92/8)	23/3 (88/12)	0.68	—	—
Mean ALT quotient (SD)	7.4 (6.6)	6.9 (8.6)	0.89	—	—	3.2 (3.3)	3.7 (3.1)	0.50	—	—
HIV risk behavior (MSM vs. non—MSM)	19/1 (95/5)	3/1 (75/25)	0.31	—	—	48/18 (73/27)	14/12 (54/46)	0.09	0.93 (0.21–4.06)	0.92
On HAART	20 (100)	3 (75)	0.17	—	—	60 (91)	23 (88)	0.71	—	—
HIV—RNA <50 copies/mL	16 (80)	2 (50)	0.25	—	—	47 (71)	11 (42)	0.02	2.13 (0.86–4.13)	0.28
Mean CD4 cell count (SD), 10^6^ cells/L	500 (102)	461 (125)	0.51	—	—	443 (87)	389 (68)	0.006	1.01 (0.99–1.02)	0.17
HCV RNA (SD), log_10_ IU/mL	4.75 (1.44)	6.16 (0.86)	0.07	0.17 (0.02–1.64)	0.13	5.93 (1.48)	6.76 (0.75)	0.008	0.35 (0.14–0.84)	0.02
Predominant HCV genotype (1/6 vs. non-1/6)‡	9/11 (45/55)	3/1 (75/25)	0.59	—	—	36/30 (55/45)	17/9 (65/35)	0.48	—	—
*IL28B* rs8099917 genotype (TT vs. non-TT)	17/3 (85/15)	2/4 (50/50)	0.18	—	—	54/12 (82/18)	13/13 (50/50)	0.004	5.56 (2.39–8.33)	0.006
METAVIR fibrosis stage (<F2 v.s. ≥F2)¶	18/2 (90/10)	3/1 (75/25)	0.44	—	—	37/28 (57/43)	11/15 (42/58)	0.25	—	—
RVR (yes vs. no)	20/0 (100/0)	4/4 (100/0)	—	—	—	43/23 (67/23)	7/19 (27/73)	0.006	8.33 (5.89–14.3)	0.003

SD, standard deviation; ALT, alanine aminotransferase; HIV, human immunodeficiency virus; MSM, men who have sex with men; HAART, highly active antiretroviral therapy; CD, cluster of differentiation; RNA, ribonucleic acid; HCV, hepatitis C virus; *IL28B*, interleukin-28B; RVR, rapid virologic response; OR, odds ratio; CI, confidence interval.

^*^Values are numbers (percentages) unless otherwise indicated.

^†^Factors with a *p* value <0.10 in univariate analysis entered multivariate analysis.

^‡^Patients with genotype 1 or 6 monoinfection and those with genotypes 1/2, or 1/6a mixed infection were categorized as predominant HCV genotype 1/6 infections.

^¶^The transient elastography (Fibroscan®, Echosens, Paris) failed to assess the stage of hepatic fibrosis in one chronic HCV-infected patient who achieved SVR after treatment.

**Table 6:** Factors predictive of SVR in HIV-infected patients with chronic HCV-1/6 or HCV-2/3 infection.

Variables*	HCV-1/6 infection (N = 53)					HCV-2/3 infection (N = 39)				
	Univariate analysis			Multivariate analysis†		Univariate analysis			Multivariate analysis†	
	SVR (n = 36)	Non-SVR (n = 17)	*p* value	OR (95% CI)	*p* value	SVR (n = 30)	Non-SVR (n = 9)	*p* value	OR (95% CI)	*p* value
Mean age (SD), y	37(8)	45 (10)	0.005	0.88 (0.78–0.99)	0.04	35 (7)	35 (9)	0.95	—	—
Sex (male vs. female)	34/2 (94/6)	14/3 (82/18)	0.31	—	—	27/3 (90/10)	9/0 (100/0)	0.99	—	—
Mean ALT quotient (SD)	3.0 (3.5)	1.9 (1.1)	0.18	—	—	3.3 (2.7)	3.2 (3.3)	0.47	—	—
HIV risk behavior (MSM vs. non-MSM)	23/13 (64/36)	7/10 (41/59)	0.15	—	—	25/5 (83/17)	7/2 (78/22)	0.65	—	—
On HAART	34 (94)	15 (88)	0.59	—	—	26 (87)	8 (89)	0.99	—	—
HIV-RNA <50 copies/mL	27 (75)	7 (41)	0.30	—	—	20 (67)	4 (44)	0.27	—	—
Mean CD4 cell count (SD), 10^6^ cells/L	427 (70)	389 (71)	0.08	1.01 (0.99–1.02)	0.38	462 (101)	388(65)	0.05	1.01 (0.99–1.03)	0.06
HCV RNA (SD), log_10_ IU/mL	6.06 (1.25)	6.99 (0.58)	0.006	0.18 (0.03–0.98)	0.03	5.78 (1.72)	6.33 (0.86)	0.35	—	—
*IL28B* rs8099917 genotype (TT vs. non-TT)	28/8 (78/22)	8/9 (47/53)	0.03	5.52 (1.55–12.2)	0.02	26/4 (87/13)	5/4 (56/46)	0.07	2.78 (0.86–4.23)	0.07
METAVIR fibrosis stage (<F2 v.s. ≥F2)¶	20/16 (56/44)	6/11 (35/65)	0.24	—	—	17/12 (59/41)	5/4 (56/44)	0.73	—	—
RVR (yes vs. no)	20/16 (56/44)	2/15 (12/88)	0.003	9.62 (3.89–15.3)	0.007	23/7 (77/23)	5/4 (56/44)	0.24	—	—

SD, standard deviation; ALT, alanine aminotransferase; HIV, human immunodeficiency virus; MSM, men who have sex with men; HAART, highly active antiretroviral therapy; CD, cluster of differentiation; RNA, ribonucleic acid; HCV, hepatitis C virus; *IL28B*, interleukin-28B; RVR, rapid virologic response; OR, odds ratio; CI, confidence interval.

^*^Values are numbers (percentages) unless otherwise indicated.

^†^Factors with a *p* value < 0.10 by univariate analysis entered multivariate analysis.

^‡^Patients with genotype 1 or 6 monoinfection and those with genotypes 1/2, or 1/6a mixed infection were categorized as predominant HCV genotype 1/6 infections.

^¶^The transient elastography (Fibroscan®, Echosens, Paris) failed to assess the stage of hepatic fibrosis in one chronic HCV-2/3 patient who achieved SVR after treatment.
